# A profile of emergency readmissions to Department of Surgery, Victoria Hospital, Blackpool

**DOI:** 10.1186/1753-6561-6-S4-P2

**Published:** 2012-07-09

**Authors:** G Samra, K Mehta

**Affiliations:** 1 Blackpool Fylde and Wyre NHS Hospitals Trust, UK

## Introduction

For several years an increasing number of patients are being readmitted to hospital as an emergency soon after their initial discharge. The reasons behind such readmissions are highly complex, and studies have so far failed to identify the definitive drivers of this trend.

## Aims

To create a profile of emergency readmissions to the Department of Surgery, Victoria Hospital, Blackpool (June-July 2010) encompassing the following profile elements: Patient demographics, source of admission, surgical specialty involved in patient care, duration of hospital stay during admissions, gap between discharge and readmission, reason for readmission, surgical procedure and investigation during admissions.

## Methods

Data Source: Informatics Department and patient admission register on Surgical Assessment Unit (Retrospective data collection); Data Compilation: Patient Admission Database (PAD); N = 68.

## Results

Age distribution: 33.8% of patients readmitted were 41-60 years old and 29.4% were >61years old. Gender distribution: No significant difference in the gender distribution of patients readmitted. Source of first admission: 61.7% of patients readmitted were referred from GP and 33.8% of patients readmitted were referred from AE as the first source of admission. Specialty distribution: General Surgery 77%; Hepato-billiary 8%; Breast 4%; Vascular 4%; Colo-rectal 3%; Urology 4%. Duration of stay: The average duration of stay during the first admission was 5 days. The average gap between discharge and readmission was 7 days. The average duration of stay during the first admission was 10 days. Reason for readmission: Same Diagnosis 57%; Pain 18%; Bleeding 4%; Infection 6%; Constipation 4%; Nausea-vomiting 2%; Other reason 9%. Procedure on first admission (top three): 1. Appendecectomy, 2. Incision and drainage of abcess, 3. Circumscision. Procedure on second admission (top 3) 1. Catheter change, 2. Incision and drainage of abcess, 3. Cholecystectomy.

## Conclusions

Elderly patients admitted for less than a week from primary care as emergency admissions with general surgical complaints have a high risk of readmission. This study identifies patient, health-care service and disease variables that relate to high readmission rates and lays the foundation for addressing pertinent issues.

